# Bilateral adrenal neuroblastoma: peculiar pattern of a rare pediatric presentation

**DOI:** 10.1007/s12672-024-00966-6

**Published:** 2024-04-12

**Authors:** Mohamed Fawzy, Gehad Ahmed, Yasser Youssef, Naglaa Elkinaai, Amal Refaat, Mai Amr Elahmadawy, Fadwa Said, Salma Elmenawi

**Affiliations:** 1 Pediatric Oncology Department, Children’s Cancer Hospital Egypt-57357, Cairo, Egypt; 2 National Cancer Institute, Cairo University, Cairo, Egypt; 3 Faculty of Medicine, Department of General Surgery, Helwan University, Cairo, Egypt; 4 Surgical Oncology Department, Children’s Cancer Hospital Egypt-57357, Cairo, Egypt; 5 Pathology Department, Children’s Cancer Hospital Egypt-57357, Cairo, Egypt; 6 Radiodiagnosis Department, Children’s Cancer Hospital Egypt-57357, Cairo, Egypt; 7 Nuclear Medicine Department, National Cancer Institute, Cairo University, Cairo, Egypt; 8 Department of Clinical Pathology, Cairo University, Cairo, Egypt; 9 Clinical Research Department, Children’s Cancer Hospital Egypt-57357, 1 Seket Al-Emam Street, El-Madbah El-Kadeem Yard, El-Saida Zenab, Cairo, Egypt

**Keywords:** Neuroblastoma, Suprarenal, Bilateral, BSN, Multifocal

## Abstract

**Background:**

Bilateral suprarenal neuroblastoma (BSN) is a rare presentation. Few previously published literature showed BSN patients to have favorable pattern and prognosis. This study aim was to evaluate clinical and biological features in relation to outcome of Egyptian patients with BSN.

**Methods:**

Included patients were diagnosed from 2007 to 2017, retrospectively. Tissue biopsy, imaging and bone marrow were evaluated at presentation. Clinical, demographic, biological variables and risk group were determined and analyzed in relation to overall (OS) and event-free-survival (EFS).

**Results:**

BSN patients (n = 33) represented 2% of hospital patients with neuroblastoma during the 10-year study period, 17 were males and 16 were females. Twenty-four patients (72.7%) were infants, and 9 patients (27.3%) were above 1 year of age (range: 1 month to 3 years). Metachronous disease was present in only one patient. Amplified MYCN was found in 10 patients. Initially, most patients (n = 25) had distant metastasis, 6 had stage 3 versus 2 stage 2. Fifteen were high risk (HR), 15 intermediate (IR), 1 low risk (LR) and 2 were undetermined due to inadequate tissue biopsy. Three-year OS for HR and IR patients were 40.5% and 83.9% versus 23.2% and 56.6% EFS; respectively.

**Conclusion:**

BSN treatment is similar to unilateral disease. A more conservative surgical approach with adrenal tissue preservation on less extensive side should be considered. Biological variables and extent of disease are amongst the most important prognostic determinants. Future studies are warranted to further address the biologic profiling of BSN and highlight prognostic significance of size difference between both adrenal sides.

## Introduction

Neuroblastoma is the most common extracranial solid tumor occurring in childhood with diverse clinical presentation and course depending on tumor biology. Unique features of such neuroendocrine tumors are the early age of onset, high frequency of metastatic disease at diagnosis and the tendency for spontaneous regression of tumors presenting during infancy [1]. While the most common site of origin is the adrenal gland, yet bilateral adrenal neuroblastoma is rare, it can be due to either multifocal primary or contralateral metastasis [2]. Nickerson et al. reported that 5 out of 80 cases (6%) with stage 4S disease had bilateral adrenal neuroblastoma while in a previous study its incidence was as high as 14% among stage 4S patients (13/94) [3]. The majority of bilateral adrenal neuroblastoma occur under 6 months of age at diagnosis and carry a favorable prognosis in most of the cases [4]. Other investigators reported bilateral adrenal neuroblastoma in an 8-year-old child [5]. Clinical behavior of bilateral suprarenal neuroblastoma is exceptional with several multicystic forms, variable MYCN amplification, widespread metastases and high mortality [2].

Cases exhibiting risk factors, such as amplified MYCN, show comparable results to high-risk unilateral neuroblastoma carrying the same prognostic features.

Both low incidence of relapse and the risk of adrenal failure associated with radical surgery, argue against an aggressive surgical approach in patients with BSN [6]. Bilateral adrenalectomy is sometimes unavoidable, thus unilateral excision with contralateral enucleation or partial resection/observation could be adopted [2]. The aim of the present study was to find out disease pattern, management and outcome of newly diagnosed patients with BSN.

## Patients and methods

Study included patients with BSN presented to Children’s Cancer Hospital-Egypt (CCHE) during the period from July 2007 to December 2017. Treatment plan was approved by Children’s Cancer Hospital 57357-Egypt Institutional Review Board and informed consents were obtained from all study patients (or from legal guardians in the case of children under 16). The study was performed following the ethical standards as laid down in the 1964 Declaration of Helsinki and its later amendments.

All demographic and clinical data were retrospectively extracted from patients’ medical records including initial presentation, checkpoint evaluation as per treatment protocols, disease response according to international neuroblastoma response criteria [7, 8].

Diagnosis of NB was carried out by ultrasound/CT-guided biopsy taking of the primary tumor from at least one side, according to the International Neuroblastoma Pathology Classification (INPC) [9, 10]. Further biological work-up was completed on fresh tissue specimens that included MYCN gene amplification for all study patients and DNA ploidy for patients under 18 months of age. Other investigations, CT/ MRI of the primary tumor, metastatic workup in the form of bilateral bone marrow aspirates and biopsy, Tcm99 bone scan, and PET-CT/MIBG scan were done.

Risk stratification COG system was applied, based on patient’s age, INSS stage, INPC, DNA ploidy and MYCN status [11–13].

Low risk patients are routinely treated by upfront excision followed by periodic observation. Wait and see strategy was adopted in some asymptomatic, uncomplicated low risk stage 1, 2 and 4 s patients.

Till 2012, newly diagnosed patients with IR NB were treated with OPEC/OJEC regimen, whereas high risk patients received SFOP NBL90 protocol [14, 15]. Starting in January 2013, treatment protocols were switched to SFOP NBL90 protocol for intermediate risk patients and COG A3973 for high risk patients [14, 16].

Treatment regimen for HR patients is composed of systemic induction chemotherapy, local control (surgical resection of primary tumor) and HSCT consolidation followed by radiation of local and active metastatic sites and retinoid therapy (13 cis RA) for 6 months. Maintenance chemotherapy courses were administered while waiting for HSCT (COG A-3973 protocol). Hematopoietic stem cell harvesting was usually carried out after 2–3 cycles of induction chemotherapy. COG-ANBL00P3 protocol of opsoclonus myoclonus ataxia syndrome (OMAS) was used for candidate patients(s) [17].

Patients undergone bilateral adrenalectomy received hormonal replacement therapy in form of hydrocortisone or adrenocorticotropic hormone.

### Statistical analysis

Data were retrieved from patients’ paper and electronic medical records. Statistical analysis was done using SPSS (Statistical Package for the Social Science; IBM, USA) version 20 for Microsoft Windows and R statistical environment (version 3.4.0), using R packages survival (version 2.41-3), Survminer (version 0.4.2), as well as ggplot2 (version 2.2.1). Median and range were used to express quantitative data, while qualitative data were expressed as frequency and percentage. Kaplan Meier was used for estimation of overall and event-free survivals (OS, EFS); comparison was done by log-rank test. In case of EFS, time to event was time from enrollment till time of recurrence/progression/death due to any cause, or till date of last contact, if no event occurred; as for OS, time to event was time from enrollment till death due to any cause or till date of last contact, if no death occurred. Survival estimates were reported as 5-year survival ± SE (standard error). A p-value of less than 0.05 was considered statistically significant.

## Results

Thirty-three patients with bilateral adrenal NB were included; 16 males and 17 females (M/F = 0.94:1). Around 70% of study patients were less than 1 year, 3% between 1 and 1.5 and 27% above 1.5 years old at presentation (median: 0.5 years, range: 0.1–2.7 years). Most of patients (25/33; 76%) presented with metastatic disease (20 stage 4 and 5 stage 4 s), 8/33 (24%) had loco-regional disease (7 stage 3 and 1 stage 2). No adrenal masses were detected by prenatal testing in any of these children. Unfavourable INPC was found in 82% while one third of specimens showed amplified MYCN. Each of IR and HR groups constituted 45.5% (15 patients each) of all patients versus only 1 LR patient (3%) and undetermined risk in 2 patients (6.1%) due to inadequate tissue biopsy, (Table 1).

**Table 1:** Characteristics of study cohort

	Frequency (n = 33)	Percent (%)
Gender		
Female	17	51.5
Male	16	48.5
Age		
Less than 1 year	24	70.6
1 to less than 1.5 yrs	1	2.9
1.5 to 5 yrs	8	26.5
Onset		
Metachronous	1	3
Synchronous	32	97
Presenting manifestations^a^		
Swelling & abdominal distension	26	79
Anemia	8	24
Bleeding	3	9
Jaundice	1	3
OMAS^b^	1	3
Survival status		
Alive	17	57.6
Dead^c^	14	42.4
Relapse/progression status		
Yes	11	36.4
No	22	63.6
INSS stage		
1	0	0
2	2	6.0
3	6	18.2
4	20	60.6
4s	5	15.2
MYCN		
Amplified	10	30.3
Not amplified	18	54.5
Unknown	5	15.2
INPC		
Favorable	2	6.1
Unfavorable	27	81.8
Unknown	4	12.1
Surgery		
Done	8	24.2
Unilateral	4	
Bilateral	4	
Not done	25	75.8
Risk		
High risk	15	45.5
Intermediate risk	15	45.5
Low risk	1	3.0
Undetermined^c^	2	6.1
Regimen		
SFOP NBL90	14	42.4
COG A3793	10	30.3
No chemotherapy	6	18.2
COG ANBL00P3 (OMAS) protocol	1	3.0
OPEC/OJEC	2	6.1
BMT^d^		
No	30	90.9
Yes	3	9.1

^a^some patients had more than 1 manifestation

^b^ OMAS: opsoclonus myoclonus ataxia syndrome

^c^2/14 died after less than 2 weeks of diagnosis without receiving treatment (excluded from survival analysis)

^d^BMT: Bone Marrow Transplant

**Fig. 1:**
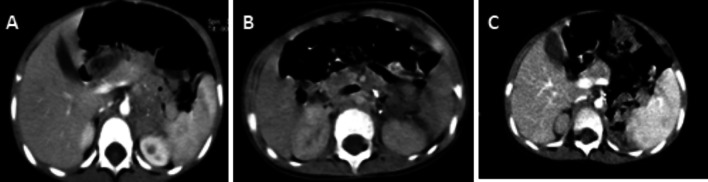
Images from the case with metachronus bilateral adrenal neuroblastoma; **A** left adrenal mass at presentation; **B** transverse section after left adrenalectomy; **C** newly developed small right adrenal mass

Surgery was done in 8 patients; 4 unilateral adrenalectomy with contralateral small post-chemotherapy residual, the other 4 patients undergone bilateral adrenalectomy. One of the 4 patients with bilateral adrenalectomy had metachronous onset of disease. Patient presented at the age of 2.6 years with left adrenal mass associated with OMAS manifestations; she was treated with steroids for the OMAS symptoms and had complete resection of the left adrenal mass. Sixteen months later, same patient developed new tumor on the contralateral adrenal gland that was also resected (Fig. 1). Only 4 patients received radiotherapy; 3/15 high risk patients took radiotherapy on primary and distant sites with most active lesions after having BMT; 1/15 HR patients took radiotherapy on primary site and didn’t receive BMT.

Two out of the 33 study patients died within one week of presentation before receiving any treatment (both had poor general condition due to advanced disease) and excluded from survival analysis.

Twelve patients (36.4%) witnessed either relapse or progression of their disease during study period (9 local, 2 metastatic and 1 combined local and distant metastatic disease progression).

Disease unrelated mortality was reported in 5/14 patients, either due to severe infection or adrenal insufficiency.

**Fig. 2:**
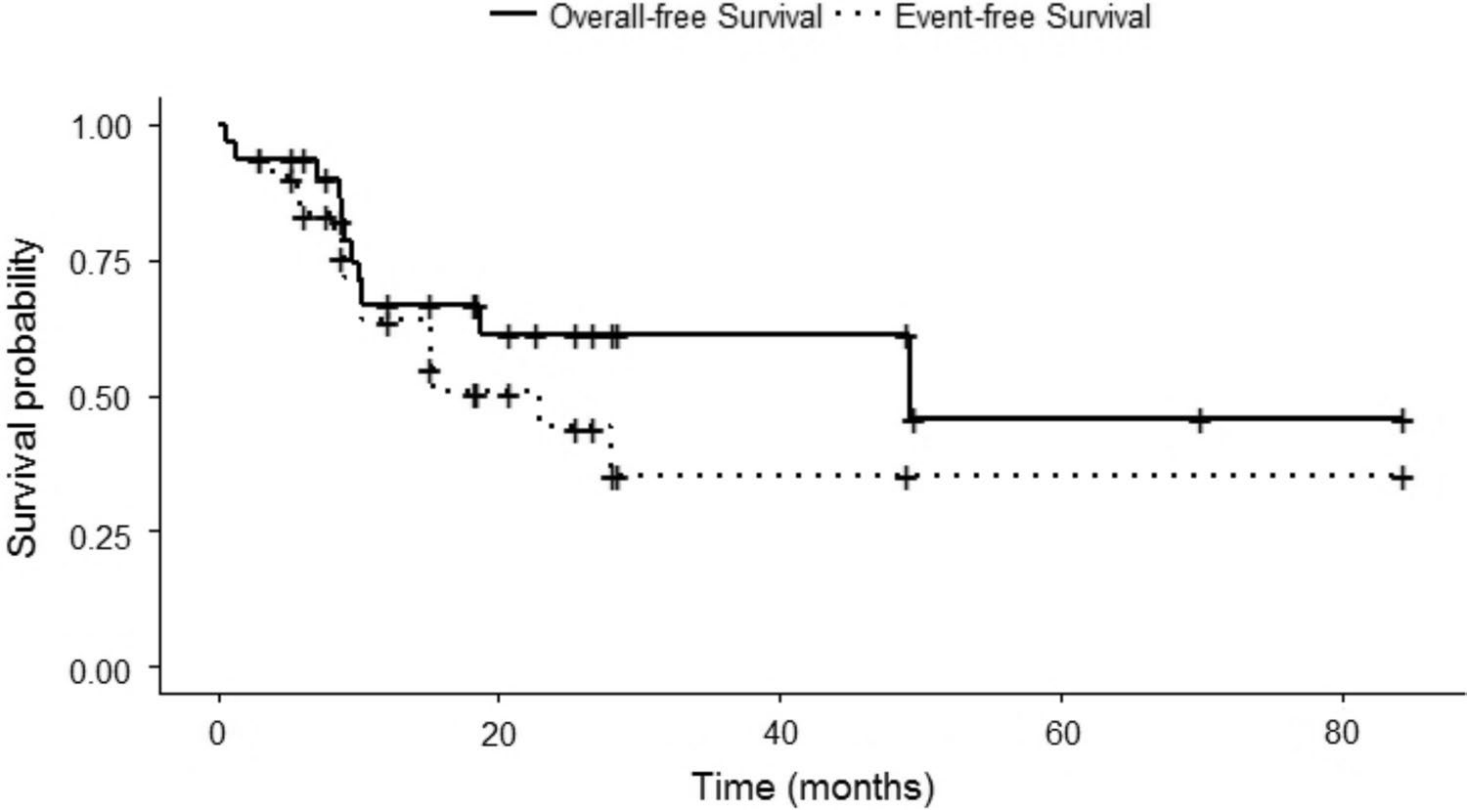
Overall and event-free survival of study cohort

The 5-year OS and EFS for eligible study cohort (n = 31) were 45.9 ± 15.2% and 35.4 ± 11.6%, respectively, with median follow-up of 19 months (range: 0.5–103 months), (Fig. 2).

Survival analysis by risk group showed higher 3-year OS & EFS rates for IR patients (OS = 83.9 ± 10.4% and EFS = 56.6 ± 18.8%) compared to HR patients (OS = 40.5 ± 14.7% and EFS = 23.2 ± 13.2%).

Tumor size and whether the difference between the two adrenal sides had any prognostic impact on disease outcome was investigated in this study. Size of all tumors ranged from 1.5 to 14 cm while the median difference between right and left adrenal glands was 3.6 cm. Interestingly, 4 out of 6 patients having > 5 cm size difference in maximum diameter between right and left sides showed poorer prognosis in the form of either disease relapse or progression, whereas 5 out of another 6 patients with a difference < 1 cm between both sides showed better outcome, i.e. no relapse, progression or death, (Table 2).

**Table 2:** Characteristics of patients with events (relapse/progression/death)

Age (years)	Gender (m/f)^a^	INSS Stage	MYCN^b^	INPC^c^	Risk^d^	regimen	Size difference between both adrenal glands (cm)^e^	BMT	Relapse/progression	Time till relapse/progression (months)	Alive/dead (reason)^f^	Time from diagnosis till death (months)
1.3	m	3	A	UH	HR	SFOP	8	No	Yes	5.72	Dead (dr)	8.72
0.5	f	4	A	UH	HR	SFOP	8.8	Yes	Yes	28.03	Dead (dr)	49.21
0.2	m	4	A	UH	HR	SFOP	2.8	No	Yes	5.92	Dead (dr)	7.14
0.9	f	4	A	UH	HR	A3973	1	No	Yes	8.26	Dead (dr)	10.03
0.4	f	4	A	UH	HR	A3973	3.8	No	No	–	Dead (encephalopathy due to status epilepticus)	10.2
2.1	m	4	A	UH	HR	A3973	6.1	No	Yes	4.01	Dead (dr)	9.51
0.2	f	4	A	UH	HR	SFOP	3.5	No	Yes	15.1	Dead (dr)	18.65
1.9	m	4	A	UH	HR	A3973	4.2	No	Yes	15.1	Dead (dr)	18.82
1.6	m	4	Not done	UH	HR	A3973	na^e^	No	No	–	Dead (chest infection)	1.25
1.8	m	4	NA	UH	HR	A3973	na^e^	No	Yes	9.44	Alive	–
1.6	m	3	NA	UH	HR	A3973	8.8	No	Yes	7.73	Alive	–
0.4	m	4	NA	UH	IR	SFOP	5	No	No	–	Dead (adrenal failure)	8.78
1	f	4	NA	UH	IR	SFOP	na^e^	No	No	–	Dead (renal tubular dysfunction)	9.08
1.8	f	4	NA	UH	HR	A3973	na^e^	No	Yes	9.44	Alive	–
1.6	m	3	A	UH	HR	A3973	na^e^	No	Yes	7.73	Alive	–

^a^m: male; f: female

^b^A: amplified, NA: not amplified;

^c^ UH: unfavorable histology

^d^risk: HR, high risk; IR, intermediate risk

^e^na: not applicable, since one or both adrenal glands were forming mass nodal complex

^f^dr: disease-related death

## Discussion

Near half of NB tumors arise from one of the adrenal glands [18], whereas BSN is rare entity that can be due to multifocal primary or contralateral metastasis that usually presents at younger age [2]. Familial NB typically diagnosed in early age and can present with multiple primary tumors as well with family history present in 1–2% of NB cases. Germline ALK mutations could explain most of the hereditary NB, while less frequently, somatically acquired mutations may occur [19]. Other genetic abnormalities as PHOX2B deletion and NF1 mutation were also reported in cases with familial NB [20]. Due to small numbers, no prospective studies were systematically conducted on BSN, yet only case reports and retrospective data were published.

In CCHE, 1606 patients with newly diagnosed NB were reviewed where only 2% (n = 33) reported to have bilateral adrenal primary site of either simultaneous or metachronous onset. More than 70% of the present study patients aged below 1 year (median: 6 months), in accordance to most of the previously published data. Very similarly, reviewed SEER data of 1617 patients between 1973 and 2012 revealed that BSN constituted 2% (n = 32) of all patients. Significantly greater proportion of patients with BSN had distant metastasis at diagnosis (90.6% vs. 69.0%; P = 0.006) and 53.1% were below 1 year of age (P = 0.01), [6, 18]. On the other hand, higher incidence of 6% and above was reported [3, 21].

Compared to unilateral disease, BSN more likely present with distant metastasis but more favorable outcome [6, 18]. Two retrospective studies included 15 and 45 BSN cases respectively reported median age of 4 months and 3 months at diagnosis and similar percentages of infants (86.6%) [6].

On contrary, a brief report published in 2001 by Cunsolo et al. showed a case of BSN presented in of 7.5 years old boy who suffered an aggressive disease and witnessed 2 recurrences later on, ended by death after 2 years from presentation [22]. Another letter to the editor in 2021 reported a 3.8-year-old child with advanced BSN and blindness as a manifestation of disease metastasis [23].

The 5-year OS rate was 90% for stage 4S patients with BSN [6], and 70.5% in another study, compared to 62.4% for unilateral disease [18]. Widespread metastases, higher mortality rates and variable MYCN amplification status were generally reported in another cohort [2]. Similar to HR unilateral NB, only BSN cases showing same poor prognostic features will carry unfavourable prognosis [6].

In the present study, aggressive disease of BSN of less favourable prognosis was reported with 5-year OS was 45.9%. Yet, we don’t believe that low survival rate can be attributed to any BSN specific variables. Noticed poorer OS could rather be explained by the unfavourable underlying disease features; advanced stage 4 disease encountered in 20 patients (60.6%), amplified MYCN in 30%, unfavourable INPC in 81% and 45.5% of all patients had HR disease with 63.6% witnessed either relapse or progression. Older patients age (30% of study group) and disease unrelated deaths (5 out of 14) had an additional negative prognostic impact. The lack of detailed biological features in previous studies (case reports/series or population-based registries) can explain the diverse outcome between our results and previously published data.

Treatment options for BSN are generally similar to those applied in unilateral disease management; based on multidisciplinary approach and risk stratification treatment strategy except for surgery. Aggressive surgical approach is might not be recommended due to low incidence of relapse in most of cases and to try minimizing the risk of adrenal failure following bilateral adrenalectomy [6].

The main goal of surgery in patients with NB should target most complete resection of the mass (achieving > 90% resection), preserving surrounding structures [24]. Adrenal sparing surgery on one side has been described with good outcome in BSN. This technique feasibility mostly depends on the presence of some normal-looking adrenal tissue and the chance to preserve at least one vein and one artery [25].

Size of the primary adrenal tumor at presentation and the difference between both right and left sides was investigated in this cohort. Interestingly, tumors with ≥ 5 cm difference of maximum diameter between two sides carried poorer prognosis compared to others. This unprecedented finding could be included in future studies as one of potential prognostic indicators that worth further investigation.

## Conclusion

Bilateral suprarenal neuroblastoma is a rare presentation mostly occurring in infancy, its treatment is similar to unilateral disease. More conservative surgical approach with adrenal tissue preservation on less extensive side should be considered whenever feasible. Similar to unilateral disease, biological variables and extent of disease at presentation are amongst the most important prognostic determinants with currently no specific or unique underlying variables confirmed to be peculiar to BSN. Future studies are warranted to further address biologic profiling of BSN and highlight the prognostic significance of size difference between tumors on both adrenal sides.

## Data Availability

The datasets generated during and/or analyzed during the current study are available from the corresponding author on reasonable request.
